# Trifluridine/Tipiracil in the Real-World Management of Metastatic Gastric and Gastroesophageal Junction Cancers in Canada

**DOI:** 10.3390/curroncol30010011

**Published:** 2022-12-22

**Authors:** Philip Q. Ding, Aastha Dolley, Winson Y. Cheung

**Affiliations:** 1 Oncology Outcomes, Calgary, AB T2N 4Z6, Canada; 2 Faculty of Medicine & Dentistry, University of Alberta, Edmonton, AB T6G 2R7, Canada; 3 Taiho Pharma Canada, Inc., Oakville, ON L6H 5R7, Canada; 4 Department of Oncology, Cumming School of Medicine, University of Calgary, Calgary, AB T2N 4N2, Canada

**Keywords:** gastric cancer, gastroesophageal junction, real-world evidence, chemotherapy, treatment patterns, patient support programs

## Abstract

Background: Gastric cancer mortality remains among the highest of all cancers. Trifluridine/tipiracil (FTD/TPI) represents Canada’s first standard-of-care, third-line, systemic therapy for metastatic gastric/gastroesophageal cancer. We characterized real-world treatment patterns in patients enrolled to receive FTD/TPI through Taiho Pharma Canada’s Patient Support Program. Methods: Demographic and clinical information were collected from November 2019 to November 2021 for adult patients with refractory metastatic gastric/gastroesophageal cancer throughout Canada. We examined all variables using descriptive statistics and performed survival and association analyses. Results: 162 patients enrolled to receive FTD/TPI with a median age of 65 years, 12 of whom had HER2 positive disease. Among 123 patients who started FTD/TPI, median follow-up was 3.1 months and median progression-free survival (PFS) was 3.5 months (95% CI 3.2–4.0). Among 121 patients who discontinued FTD/TPI, median treatment duration was 2.39 cycles (IQR 1.14–3.86). A total of 52% discontinued treatment due to disease progression, and 27% had a dose reduction or delay. On multivariable logistic regression, prior FOLFIRI was a statistically significant predictor of treatment modification. Conclusions: Through the Patient Support Program, FTD/TPI is an actively utilized treatment option in heavily pretreated metastatic gastric/gastroesophageal cancer, despite its recent introduction. With longer-than-expected treatment duration and PFS, FTD/TPI likely addresses an important unmet need for effective and tolerable therapies in this setting.

## 1. Introduction

Cancers of the stomach and esophagus were responsible for an estimated 1.7 million new cancer diagnoses and 1.3 million deaths in 2020 worldwide [1]. The Canadian Cancer Society projected 6400 new cases of gastric and esophageal cancers in Canada within 2021 [2]. Mortality from gastric and esophageal malignancy remains among the highest of all cancers, despite declining trends, with estimated five-year survival rates of 29% and 16% in Canada, respectively [2]. Gastroesophageal junction cancers are generally classified according to the American Joint Committee on Cancer as either gastric or esophageal cancer, depending on the location of its epicentre relative to the cardia [3]. In the locally advanced and metastatic settings, however, gastric and gastroesophageal junction cancers are managed similarly [4,5].

Palliative care is central to the treatment of patients with metastatic gastric/gastroesophageal cancer, including gastroesophageal junction adenocarcinoma, and should involve a multimodality interdisciplinary approach with symptom control, psychosocial, and spiritual interventions [4]. For patients with an adequate performance status (i.e., Karnofsky score ≥ 60% or ECOG score ≤ 2), systemic therapy may be used alongside best supportive care as a life-extending measure [4,6,7]. The most common first-line treatments in Canada include fluoropyrimidine/platinum combinations or fluoropyrimidine/irinotecan combinations [4,6,8]. Nivolumab and pembrolizumab have also been approved in Canada for HER2-negative disease, in combination with fluoropyrimidine/platinum chemotherapy [9,10]. In patients with HER2-positive disease, the addition of trastuzumab to cisplatin and a fluoropyrimidine is a standard practice that has proven survival benefits [4,6,11]. Second-line therapies are largely dependent on both prior drugs and performance status and may include combination (e.g., fluoropyrimidine/platinum, fluoropyrimidine/irinotecan, and ramucirumab/paclitaxel) or single agent (e.g., irinotecan, docetaxel, paclitaxel, and ramucirumab) therapies [4,6,12]. For each line of therapy, differential funding and reimbursement between drugs remains a major determinant of regimen selection.

Trifluridine/tipiracil (FTD/TPI, TAS-102, LONSURF) received approval from Health Canada on 19 November 2019, as a third-line therapy for patients with metastatic gastric cancer, including gastroesophageal junction adenocarcinoma, who have been previously treated with at least two lines of chemotherapy. FTD/TPI is an orally-administered combination chemotherapeutic agent comprised of an antineoplastic thymidine-based nucleoside analogue and a thymidine phosphorylase inhibitor [13]. The recommended starting dose is 35 mg/m^2^/dose twice daily on days 1 to 5 and days 8 to 12 of each 28-day cycle. The treatment cycle may be repeated until a benefit is no longer observed or unacceptable toxicity occurs [13]. FTD/TPI was first introduced in Canada in January 2018 for the treatment of refractory metastatic colorectal cancer. Following its approval for metastatic gastric/gastroesophageal cancer in Canada, FTD/TPI was first made available through the Taiho Pharma Canada Patient Support Program, which provides financial support to patients who are uninsured or underinsured, with limited financial resources. However, since 2021, trifluridine/tipiracil has been fully reimbursed and listed in almost all provinces across Canada.

Prior to FTD/TPI, there was no evidence-based, standard-of-care, third-line, systemic therapy for metastatic gastric/gastroesophageal cancer in Canada [14,15,16], although certain agents were approved for third-line treatment in other countries: apatinib in China, pembrolizumab in the USA for specific indications, and nivolumab in Japan. In the pivotal 2018 phase III TAGS trial comparing FTD/TPI with best supportive care versus placebo with best supportive care in 507 patients with heavily pre-treated metastatic gastric/gastroesophageal cancer, FTD/TPI conferred significant improvements in OS and PFS, with a safety profile comparable to prior studies in metastatic colorectal cancer; the adverse events were manageable, with the most common being anemia and neutropenia [17]. Patient quality of life was maintained in the TAGS study, and compared to placebo, FTD/TPI reduced the risk of quality of life deterioration [18]. Based on data from TAGS, the pan-Canadian Oncology Drug Review issued a positive recommendation for FTD/TPI, citing a net clinical benefit that should be generalizable to the population of Canadian patients with metastatic gastric/gastroesophageal cancer [19]. The use of FTD/TPI as third-line or subsequent therapy for metastatic gastric and gastroesophageal cancers is currently endorsed in National Comprehensive Cancer Network (NCCN) and National Institute for Health and Care Excellence (NICE) guidelines [4,20,21]. However, to our knowledge, the use of FTD/TPI in real-world patients with metastatic gastric/gastroesophageal cancer in Canada has not yet been evaluated, and this is the first study to support its effectiveness and safety in routine clinical practice.

Using real-world data from the population of Canadian patients with refractory metastatic gastric/gastroesophageal cancer who enrolled to receive FTD/TPI through Taiho Pharma Canada’s Patient Support Program, we endeavoured to characterize current practice patterns in this evolving treatment landscape. We primarily aimed to describe the baseline and treatment characteristics of patients enrolled in the Patient Support Program to receive FTD/TPI for metastatic gastric/gastroesophageal cancer. Our secondary objectives were to assess survival outcomes and identify factors associated with treatment duration, treatment discontinuation, and dose modification.

## 2. Methods

### 2.1. Study Design and Population

This was a population-based, retrospective, cohort study of Canadian patients who enrolled in Taiho Pharma Canada’s Patient Support Program to receive FTD/TPI for the third- or subsequent-line treatment of metastatic gastric cancer, including gastroesophageal junction adenocarcinoma. Patients were eligible for this program if they were adults with metastatic gastric or gastroesophageal cancers who had progressed on, or were not candidates for, available chemotherapies, including a platinum, a fluoropyrimidine, and either a taxane or irinotecan, and with HER2/neu-targeted therapy if appropriate. Approval for this study was obtained from both Taiho Pharma Canada and the Health Research Ethics Board of Alberta Cancer Committee.

### 2.2. Study Data

Study data consisted of all variables available through the Patient Support Program and were provided by Taiho Pharma Canada through Bayshore HealthCare. Baseline demographic and clinical data points were collected during patient enrollment and included age, province, primary diagnosis, HER2 status, and prior therapies. Treatment data were prospectively collected for all enrolled patients, comprising enrollment date, treatment status, reason for status, treatment start date, treatment stop date, reimbursement information, body surface area, treatment dose, and treatment modification. Reasons for treatment discontinuation were assessed as per the treating physician’s discretion. Data collection proceeded from the FTD/TPI approval date of 21 November 2019, up to and including the data cut-off date of 30 November 2021.

### 2.3. Statistical Analysis

Baseline and treatment characteristics were summarized by descriptive statistics. Categorical variables were presented as frequencies and percentages, while continuous variables were expressed as median with interquartile range.

The Kaplan–Meier method was used to estimate progression-free survival (PFS) among patients who started FTD/TPI. Survival analysis utilized time-to-event data comprising the time from date of enrollment to either date of event (i.e., treatment discontinuation due to death or disease progression) or date of censoring, whichever was earliest per patient.

For patients who discontinued treatment, characteristics were compared between treatment duration subgroups using the Wilcoxon rank-sum test for continuous variables and Pearson’s Chi-squared test or Fisher’s exact test for categorical variables. Treatment duration was expressed as number of FTD/TPI cycles, with each cycle defined as 28 days of treatment [13]. The distribution of treatment duration was illustrated graphically. Multivariable logistic regression analysis was performed to identify baseline factors associated with longer treatment duration, treatment discontinuation, and dose modification, among a select array of demographic and clinical characteristics. Factors included in the multivariable analyses were those with sample size > 100 and all subgroup sizes > 10 [22].

All statistical tests were two-sided, and the significance level was defined a priori as <0.05. All analyses were performed using R (RStudio, PBC, Boston, MA, USA) [23].

## 3. Results

**Figure 1:**
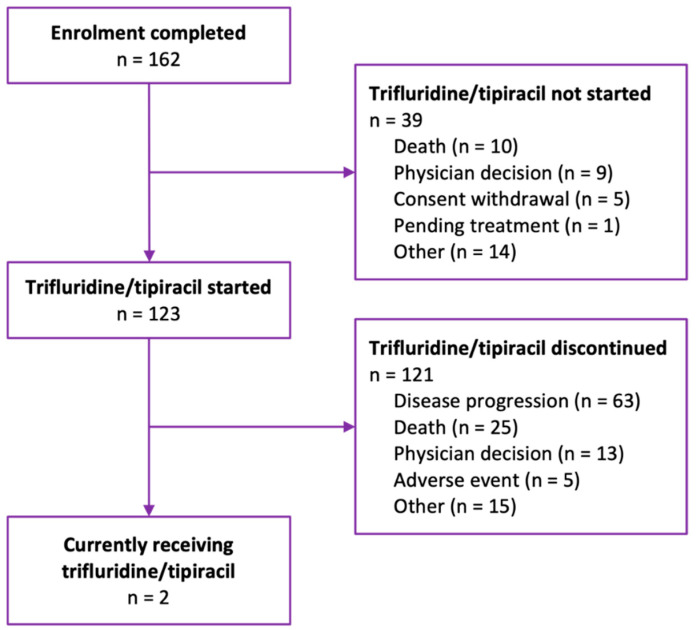
Flow diagram of patients enrolled in Taiho Pharma Canada’s Patient Support Program to receive trifluridine/tipiracil for metastatic gastric/gastroesophageal cancer.

A total of 162 patients with metastatic gastric/gastroesophageal cancer were enrolled in the Taiho Pharma Canada Patient Support Program to receive FTD/TPI (Figure 1). Among all patients enrolled, the median age at enrollment was 65 years (Table 1). Examining patient enrollment by province and territory, the greatest number of patients were from Ontario (n = 63, 38.9%). Other patients were distributed geographically as follows: 28.4% from Québec; 14.8% from British Columbia; 10.5% from Alberta, Saskatchewan, or Manitoba (Prairie Provinces); and 7.4% from New Brunswick, Newfoundland and Labrador, or Nova Scotia (Atlantic Provinces). No patients were enrolled from Prince Edward Island, the Northwest Territories, Nunavut, or Yukon. A total of 22.2% of patients (n = 36) were diagnosed with adenocarcinoma of the gastroesophageal junction. HER2 status was captured for 39.5% (n = 64) of patients, most of whom (81.2%, n = 52) had HER2 negative disease. Prior to FTD/TPI treatment, more patients received FOLFIRI (leucovorin/fluorouracil/irinotecan), FOLFOX (leucovorin/fluorouracil/oxaliplatin), paclitaxel, or ramucirumab than any other systemic therapy regimen.

**Figure 2:**
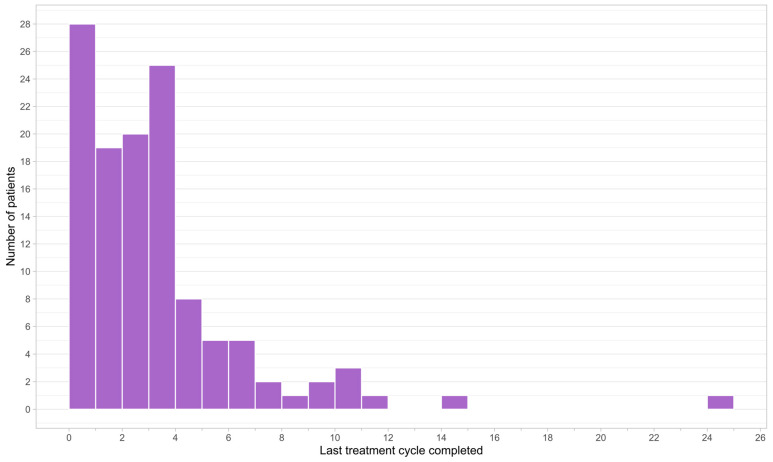
Frequency histogram of treatment duration of patients who received trifluridine/tipiracil.

At the data cut-off date and among the patients enrolled, 75.9% (n = 123) received or were currently receiving trifluridine/tipiracil. As expected in this patient population in the metastatic setting, of the patients who received FTD/TPI, 98.4% (n = 121) eventually discontinued treatment, whereas a minority (1.6%, n = 2) were still on treatment. The distribution of FTD/TPI treatment duration was right-skewed, with the majority of patients receiving less than four cycles of therapy prior to treatment discontinuation (Figure 2).

The primary reasons for never having started FTD/TPI treatment at the data cut-off date were death (25.6%, n = 10) and physician decision (23.1%, n = 9) (Table 2). For patients who started FTD/TPI, the primary reasons for treatment discontinuation were disease progression (52.1%, n = 63) and death (20.7%, n = 25). A total of 3.7% (n = 6) of patients were also enrolled in the Health Canada Special Access Program. Almost half (43.9%, n = 69) of patients received FTD/TPI treatments that were reimbursed by a compassionate access program funded by Taiho Pharma Canada. Public reimbursement was generally more rapid than private reimbursement, with a median time from enrollment of 3 days versus 7 days for private. The median duration of FTD/TPI treatment was 2.39 cycles, from treatment initiation to discontinuation. Among the patients who started FTD/TPI, 73.2% (n = 90) had their dose maintained until the end of the study period, but when modifications were employed, dose delay was more common than dose reduction.

**Figure 3:**
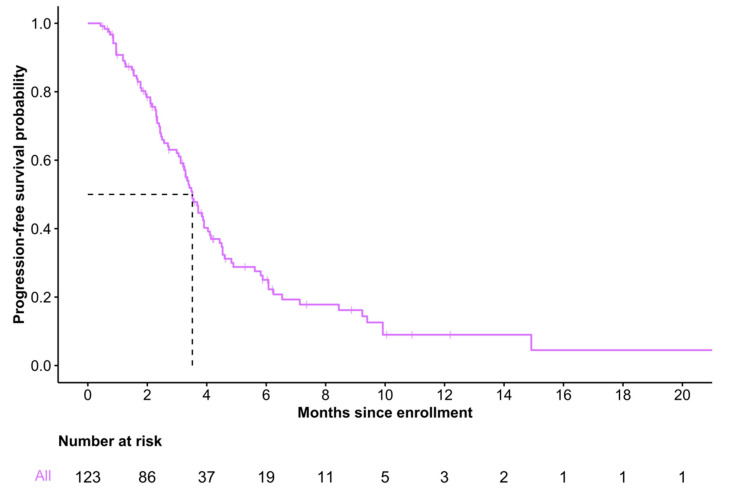
Progression-free survival of patients who initiated trifluridine/tipiracil.

Among the 123 patients who initiated FTD/TPI, median follow-up time was 3.1 months (IQR 1.7–4.5) from enrollment to treatment discontinuation. Median PFS was 3.5 months (95% CI 3.2–4.0), and 6-month PFS was estimated to be 0.25 (95% CI 0.18–0.36) (Figure 3). A total of 19 patients (15.4%) and 3 patients (2.4%) were alive without disease progression at 6 months and 1 year, respectively. At data cut-off, 88 (71.5%) of 123 patients had a disease progression or died.

Treatment discontinuation due to disease progression or physician decision occurred more frequently among patients who received at least two cycles of FTD/TPI, whereas treatment discontinuation due to adverse events was more frequent among patients receiving less than two cycles (Table 3). Other differences between treatment duration subgroups showed no statistical significance.

Of the 121 patients for whom FTD/TPI treatment duration was computed, none of the factors analyzed (i.e., age, geographic location, primary disease site, major prior therapies) were associated with longer treatment (Table 4) or treatment discontinuation due to disease progression (Table 5) in multivariable logistic regression. However, prior FOLFIRI treatment was associated with greater odds of FTD/TPI dose reduction or delay (OR 4.42, 95% CI 1.26–17.06, *p* = 0.02) in a multivariable model with the above covariates (Table 6).

## 4. Discussion

We conducted a retrospective analysis of real-world FTD/TPI use in a pan-Canadian cohort of patients with refractory metastatic gastric/gastroesophageal cancer in the Taiho Pharma Canada Patient Support Program. There were 162 patients who had progressed on, or were not candidates for, available chemotherapies, who enrolled in the program in a short span of two years after FTD/TPI approval. Rates of enrollment across Canada were proportional to the population estimates in each province [24]. Among the patients who started FTD/TPI, the majority received treatment for longer than two cycles, and the most common reasons for discontinuation were disease progression and death. Thus, our findings demonstrate a strong demand for effective therapies in the setting of heavily pre-treated metastatic gastric/gastroesophageal cancer and an important role for the Patient Support Program in providing access to novel chemotherapeutic agents prior to public drug plan funding.

Our real-world patient cohort was largely representative of the population with gastric/gastroesophageal cancer in terms of the frequencies of gastric primary disease site (73%) and HER2 over-expression (20%) [19,25]. Moreover, the four most common systemic therapies used prior to FTD/TPI reflected the standard-of-care first-line regimens, FOLFOX and FOLFIRI, and second-line monotherapies, paclitaxel and ramucirumab [4,6]. The characteristics of our study cohort were also concordant with those of the TAGS cohort when examining common baseline factors: age, primary disease site, and HER2 status [17]. Our sample of Canadian patients who may benefit from FTD/TPI not only supports the clinical relevancy of our results, but also presents the Patient Support Program as an effective method to provide widespread access to patients in urgent need of treatment.

The median duration of treatment was 2.39 cycles (IQR 1.14–3.86 cycles), which is considerably longer than in patients receiving FTD/TPI in TAGS (6.7 weeks [IQR 5.7–16.6 weeks] or 1.68 cycles [IQR 1.43–4.15 cycles]) [17]. Similarly, the median PFS in our cohort of 3.5 months (95% CI 3.2–4.0) was significantly longer than that of the TAGS treatment arm, 2.0 months (95% CI 1.9–2.3). Such discrepancy in PFS may be explained by differences in prior lines of therapy and the extensive enrollment procedures in large clinical trials versus compassionate use programs. Meanwhile, the median PFS of our cohort was concordant with that of patients with advanced gastric/gastroesophageal cancer treated with FTD/TPI alone in recent Denmark- and Japan-based studies [26,27]. Prolonged FTD/TPI treatment durations in compassionate use programs versus clinical trial cohorts have been well-documented in metastatic colorectal cancer [28,29,30,31], and may be by virtue of the enhanced feasibility of administration in non-trial settings. Although we could not examine treatment outcomes relative to a comparator group, treatment duration and PFS in our cohort were both longer than expected and, together, represent evidence of drug effectiveness in practice.

Almost all patients (98%, n = 121) who started FTD/TPI had their treatment discontinued by the end of our study period, most commonly due to disease progression. This is consistent with general treatment patterns in this setting of advanced disease and with the FTD/TPI group in TAGS. In contrast to TAGS, however, treatment discontinuations that were prompted by adverse events were less frequent (4.1% vs. 8.8% in TAGS). None of the factors analyzed by logistic regression, including age, location, disease site, and prior regimens, were predictive of longer treatment or treatment discontinuation due to disease progression. However, treatment discontinuation due to adverse events occurred more frequently among patients receiving less than two cycles of FTD/TPI, supporting the early onset of the most common side effects reported in both phase III studies of the therapy [17,32]. Overall, FTD/TPI was well-tolerated, with adverse events as expected in this real-world setting.

Treatment modification (i.e., dose delay or dose reduction) due to any cause was only reported in 26.8% (n = 33) of patients receiving FTD/TPI, whereas in the TAGS trial, 58% had at least one treatment modification [17]. Logistic regression analysis showed that prior use of FOLFIRI (leucovorin/fluorouracil/irinotecan) was predictive of FTD/TPI treatment modification, adjusting for potentially confounding covariates. Although the mechanisms of this association are unclear, it may be at least partially explained by the duration of prior myelosuppressive chemotherapy use. The standard-of-care first-line regimens for metastatic gastric/gastroesophageal cancer, FOLFIRI and FOLFOX, have comparable survival benefits, but different toxicity profiles [33,34]. Notably, FOLFOX and other platinum-based regimens often cause severe neurotoxicity, leading to early discontinuation or unsuitability for subsequent-line therapies [35]. Thus, FOLFIRI may be preferred over FOLFOX for first-line treatment, and patients on FOLFIRI may tolerate a longer duration of myelosuppressive treatment, predisposing them to a higher risk of neutropenia and treatment modification during subsequent FTD/TPI use [13,36].

At our data cut-off date, 39 patients (24%) never received FTD/TPI, despite enrolling in the program initially. The foremost reason, death, parallels the findings of a previous study from our group on FTD/TPI use in refractory metastatic colorectal cancer [28]. It also reinforces the necessity of timely treatment for advanced gastric/gastroesophageal cancer, whether to palliate or prolong survival. While the time from referral to enrollment may have been consequential to the frequency and reasons for never starting treatment, it was not a data element collected as part of our study. The median time from enrollment to initiating treatment was relatively short at 7 days (IQR 5–13 days).

Our study should be interpreted within the context of certain inherent limitations. While the Patient Support Program was effective in administering FTD/TPI during the earliest stages of its implementation in Canada, drug accessibility through the program may not be fully representative of the setting of drug funding and reimbursement under public drug plans and private insurance providers. Additionally, for privacy reasons, we were not permitted to link other data sources, and we lacked access to certain data points (e.g., total eligible patients, sites of metastasis, adverse events, prior therapy duration) not captured as part of the program. Lastly, due to the retrospective nature of our study design, the associations we explored are subject to unmeasured confounding variables.

Our understanding of FTD/TPI in metastatic gastric/gastroesophageal cancer will benefit from further characterization of the Canadian treatment landscape and data on treatment response, survival outcomes, and adverse events beyond the scope of compassionate use programs. The multinational uptake of FTD/TPI is accompanied by growing interest in the benefits of its administration in multiagent regimens and subsequent to immunotherapy [26,37,38]—practices that also merit additional exploration and research.

## 5. Conclusions

This study demonstrates that, through the Taiho Pharma Canada Patient Support Program, FTD/TPI is an actively utilized treatment option in heavily pretreated metastatic gastric/gastroesophageal cancer, despite its recent introduction in the Canadian healthcare system. The introduction of FTD/TPI in Canada likely addresses an important unmet need for effective and tolerable therapies in this setting.

## Figures and Tables

**Table 1:** Baseline demographic and clinical characteristics of patients enrolled in Taiho Pharma Canada’s Patient Support Program to receive trifluridine/tipiracil for metastatic gastric/gastroesophageal cancer.

Baseline Characteristic	n	Overall (n = 162)
**Age at enrollment, y**	162	65 (58, 73)
≤60		54 (33.3%)
61–70		52 (32.1%)
>70		56 (34.6%)
**Geographic location**	162	
Ontario		63 (38.9%)
Québec		46 (28.4%)
British Columbia		24 (14.8%)
Prairie provinces		17 (10.5%)
Atlantic provinces		12 (7.4%)
**Primary disease site**	162	
Gastric		126 (77.8%)
Gastroesophageal junction		36 (22.2%)
**HER2 status**	64	
Negative		52 (81.2%)
Positive		12 (18.8%)
**Prior lines of therapy (denominator = 162 for each)**	162	
FOLFIRI		22 (13.6%)
FOLFOX		22 (13.6%)
Paclitaxel		22 (13.6%)
Ramucirumab		17 (10.5%)
5-FU/LV		6 (3.7%)
Capecitabine		6 (3.7%)
Cisplatin		6 (3.7%)
Trastuzumab		5 (3.1%)
CAPOX		4 (2.5%)
Irinotecan		4 (2.5%)
Platinum		3 (1.9%)
Taxane		3 (1.9%)
Fluoropyrimidine		2 (1.2%)
Docetaxel		1 (0.6%)
Regorafenib		1 (0.6%)
Other		130 (80.2%)
Naive		4 (2.5%)

Values presented as median (IQR) or n (%). FOLFIRI, leucovorin/fluorouracil/irinotecan; FOLFOX, leucovorin/fluorouracil/oxaliplatin; 5-FU/LV, fluorouracil/leucovorin; CAPOX, capecitabine/oxaliplatin.

**Table 2:** Enrollment and treatment characteristics of patients enrolled in Taiho Pharma Canada’s Patient Support Program to receive trifluridine/tipiracil for metastatic gastric/gastroesophageal cancer.

Trifluridine/Tipiracil Treatment Characteristic	n	Overall (n = 162)
**Treatment status**	162	
Discontinued		121 (74.7%)
Never received		38 (23.5%)
Currently receiving		2 (1.2%)
Pending		1 (0.6%)
**Reason for never having started treatment**	39	
Death		10 (25.6%)
Physician decision		9 (23.1%)
Consent withdrawal		5 (12.8%)
Pending		1 (2.6%)
Other		14 (35.9%)
**Reason for treatment discontinuation**	121	
Disease progression		63 (52.1%)
Death		25 (20.7%)
Physician decision		13 (10.7%)
Adverse event		5 (4.1%)
Other		15 (12.4%)
**Health Canada Special Access Program enrollment**	162	
No		156 (96.3%)
Yes		6 (3.7%)
**Reimbursement type**	157	
Compassionate		69 (43.9%)
Private		37 (23.6%)
Bridging		32 (20.4%)
Public		18 (11.5%)
Cash Paying		1 (0.6%)
**Time from enrollment to therapy start, days**	123	7 (5, 13)
**Therapy duration, cycles**	121	2.39 (1.14, 3.86)
**Last treatment cycle completed**		
<1		26 (21.5%)
1		19 (15.7%)
2		22 (18.2%)
3		24 (19.8%)
≥4		30 (24.8%)
**Body surface area, m^2^**	146	1.77 (1.66, 1.95)
**First planned trifluridine/tipiracil dose, mg**	128	60 (55, 65)
**Treatment modification**	123	
Dose maintained		90 (73.2%)
Dose delay		11 (8.9%)
Dose reduction		7 (5.7%)
Dose reduction and delay		15 (12.2%)

Values presented as n (%) or median (IQR).

**Table 3:** Characteristics of patients who have received and discontinued trifluridine/tipiracil treatment, stratified by treatment duration.

Characteristic	n	Overall (n = 121)	Last Treatment Cycle Completed		*p* Value
			<2 (n = 45)	≥2 (n = 76)	
**Age at enrollment, y**	121	65 (58, 72)	65 (55, 72)	64 (59, 73)	0.34
≤60		41 (33.9%)	16 (35.6%)	25 (32.9%)	0.95
61–70		39 (32.2%)	14 (31.1%)	25 (32.9%)	
>70		41 (33.9%)	15 (33.3%)	26 (34.2%)	
**Geographic location**	121				0.44
Ontario		44 (36.4%)	16 (35.6%)	28 (36.8%)	
Québec		39 (32.2%)	15 (33.3%)	24 (31.6%)	
British Columbia		15 (12.4%)	5 (11.1%)	10 (13.2%)	
Prairie provinces		15 (12.4%)	8 (17.8%)	7 (9.2%)	
Atlantic provinces		8 (6.6%)	1 (2.2%)	7 (9.2%)	
**Primary disease site**	121				0.29
Gastric		96 (79.3%)	38 (84.4%)	58 (76.3%)	
Gastroesophageal junction		25 (20.7%)	7 (15.6%)	18 (23.7%)	
**HER2 status**	48				0.43
Negative		40 (83.3%)	13 (76.5%)	27 (87.1%)	
Positive		8 (16.7%)	4 (23.5%)	4 (12.9%)	
**Reason for treatment discontinuation**	121				**0.04**
Disease progression		63 (52.1%)	18 (40.0%)	45 (59.2%)	
Death		25 (20.7%)	12 (26.7%)	13 (17.1%)	
Physician decision		13 (10.7%)	3 (6.7%)	10 (13.2%)	
Adverse event		5 (4.1%)	4 (8.9%)	1 (1.3%)	
Other		15 (12.4%)	8 (17.8%)	7 (9.2%)	
**Health Canada Special Access Program enrollment**	121				0.56
No		118 (97.5%)	43 (95.6%)	75 (98.7%)	
Yes		3 (2.5%)	2 (4.4%)	1 (1.3%)	
**Reimbursement type**	121				0.76
Compassionate		51 (42.1%)	20 (44.4%)	31 (40.8%)	
Private		27 (22.3%)	9 (20.0%)	18 (23.7%)	
Bridging *		27 (22.3%)	9 (20.0%)	18 (23.7%)	
Public		15 (12.4%)	6 (13.3%)	9 (11.8%)	
Cash Paying		1 (0.8%)	1 (2.2%)	0 (0.0%)	
**Time from enrollment to therapy start, days**	121	7 (5, 13)	7 (5, 14)	7 (5, 12)	0.89
**Body surface area, m^2^**	113	1.78 (1.66, 1.96)	1.74 (1.65, 1.90)	1.81 (1.69, 1.99)	0.10
**First planned trifluridine/tipiracil dose, mg**	104	60 (55, 65)	60 (55, 65)	60 (55, 65)	0.91

Values presented as median (IQR) or n (%). * Patients on bridging receive compassionate product until private or public reimbursement is initiated.

**Table 4:** Multivariable logistic regression analysis of predictors of trifluridine/tipiracil treatment of at least two cycles.

Factor	Odds Ratio (95% Confidence Interval)	*p* Value
**Age at enrollment, y**		0.81
≤60	Reference	
61–70	1.36 (0.52, 3.62)	
>70	1.08 (0.41, 2.84)	
**Geographic location**		0.27
Ontario	Reference	
Québec	0.89 (0.36, 2.24)	
British Columbia	1.02 (0.29, 3.89)	
Prairie provinces	0.41 (0.11, 1.44)	
Atlantic provinces	4.48 (0.67, 89.77)	
**Primary disease site**		0.20
Gastric	Reference	
Gastroesophageal junction	1.97 (0.71, 6.05)	
**Prior FOLFIRI**		0.33
No	Reference	
Yes	1.89 (0.53, 7.88)	
**Prior FOLFOX**		0.30
No	Reference	
Yes	0.47 (0.10, 1.96)	
**Prior paclitaxel**		0.76
No	Reference	
Yes	1.37 (0.19, 12.28)	
**Prior ramucirumab**		0.78
No	Reference	
Yes	0.74 (0.08, 5.93)	

FOLFIRI, leucovorin/fluorouracil/irinotecan; FOLFOX, leucovorin/fluorouracil/oxaliplatin.

**Table 5:** Multivariable logistic regression analysis of predictors of trifluridine/tipiracil discontinuation due to disease progression.

Factor	Odds Ratio (95% Confidence Interval)	*p* Value
**Age at enrollment, y**		0.41
≤60	Reference	
61–70	0.91 (0.36, 2.31)	
>70	1.67 (0.65, 4.40)	
**Geographic location**		0.16
Ontario	Reference	
Québec	1.45 (0.59, 3.64)	
British Columbia	1.13 (0.34, 3.89)	
Prairie provinces	0.93 (0.27, 3.22)	
Atlantic provinces	0.13 (0.01, 0.89)	
**Primary disease site**		0.53
Gastric	Reference	
Gastroesophageal junction	1.38 (0.51, 3.83)	
**Prior FOLFIRI**		0.82
No	Reference	
Yes	0.86 (0.24, 3.02)	
**Prior FOLFOX**		0.22
No	Reference	
Yes	2.44 (0.60, 11.54)	
**Prior paclitaxel**		0.50
No	Reference	
Yes	0.52 (0.07, 3.60)	
**Prior ramucirumab**		0.45
No	Reference	
Yes	2.16 (0.28, 17.79)	

FOLFIRI, leucovorin/fluorouracil/irinotecan; FOLFOX, leucovorin/fluorouracil/oxaliplatin.

**Table 6:** Multivariable logistic regression analysis of predictors of trifluridine/tipiracil dose reduction or delay.

Factor	Odds Ratio (95% Confidence Interval)	*p* Value
**Age at enrollment, y**		0.97
≤60	Reference	
61–70	1.15 (0.40, 3.38)	
>70	1.06 (0.36, 3.17)	
**Geographic location**		0.71
Ontario	Reference	
Québec	1.53 (0.57, 4.21)	
British Columbia	1.05 (0.23, 4.16)	
Prairie provinces	0.69 (0.12, 2.95)	
Atlantic provinces	0.46 (0.02, 3.34)	
**Primary disease site**		0.86
Gastric	Reference	
Gastroesophageal junction	1.10 (0.35, 3.16)	
**Prior FOLFIRI**		**0.02**
No	Reference	
Yes	4.42 (1.26, 17.06)	
**Prior FOLFOX**		0.69
No	Reference	
Yes	0.74 (0.15, 3.15)	
**Prior paclitaxel**		0.79
No	Reference	
Yes	1.31 (0.16, 10.43)	
**Prior ramucirumab**		0.93
No	Reference	
Yes	0.91 (0.09, 8.00)	

FOLFIRI, leucovorin/fluorouracil/irinotecan; FOLFOX, leucovorin/fluorouracil/oxaliplatin.

## Data Availability

Data will not be shared due to patient confidentiality, according to ethics approval for this study.
